# Genetic Markers of Methotrexate Treatment Failure in Psoriasis

**DOI:** 10.3390/jpm16010005

**Published:** 2025-12-25

**Authors:** Maria N. Vikhreva, Lavrenty G. Danilov, Andrey A. Martynov, Olga A. Levashova, Svetlana N. Tuchkova, Sherzod P. Abdullaev, Karin B. Mirzaev, Andrey S. Glotov, Oleg S. Glotov, Dmitry A. Sychev

**Affiliations:** 1 Penza Institute for Advanced Medical Education—Branch of the Federal State Budgetary Educational Institution of Further Professional Education “Russian Medical Academy of Continuous Professional Education” of the Ministry of Healthcare of the Russian Federation, 8A Stasov Str., Penza 440060, Russia; olga.lewashowa@yandex.ru; 2 Federal State Budgetary Educational Institution of Higher Education “St. Petersburg State University”, 7-9 Universitetskaya Nab., Saint Petersburg 199034, Russia; lavrentydanilov@gmail.com; 3 Federal State Budgetary Research Institution “Russian Research Center of Surgery Named After Academician B.V. Petrovsky”, Abrikosovsky Lane, 2, Moscow 119991, Russia; aamart@mail.ru (A.A.M.); svetlana.tuch1998@gmail.com (S.N.T.); karin05doc@yandex.ru (K.B.M.); dimasychev@mail.ru (D.A.S.); 4 Federal State Budgetary Educational Institution of Further Professional Education “Russian Medical Academy of Continuous Professional Education” of the Ministry of Healthcare of the Russian Federation, Barrikadnaya Str. 2/1, bld. 1, Moscow 125993, Russia; 5 Federal Scientific and Clinical Center for Infectious Diseases of the Federal Medical Biological Agency of Russian Federation, 9A Professora Popova Str., Saint Petersburg 197022, Russia; anglotov@mail.ru (A.S.G.); olglotov@mail.ru (O.S.G.); 6 Federal State Budgetary Scientific Institution “The D.O. Ott Research Institute of Obstetrics, Gynecology and Reproductology”, 3 Mendeleevskaya Line, Saint Petersburg 199034, Russia; 7 State Budgetary Healthcare Institution “Moscow Scientific and Practical Center for Laboratory Research of the Moscow Healthcare Department”, 49 Orekhovy Boulevard, bld. 1, Moscow 115580, Russia

**Keywords:** personalized medicine, drug response, pharmacogenetic markers, psoriasis, methotrexate

## Abstract

**Background:** Pharmacogenetic markers associated with the need to switch patients from methotrexate (MTX) to biologic agents in moderate-to-severe psoriasis remain insufficiently studied. The pharmacokinetics of MTX depend on the individual characteristics of the patient, as well as on the function of specific transporters and enzymes involved in its absorption, distribution, metabolism, and elimination; therefore, polymorphisms in genes encoding these proteins may be considered pharmacogenetic predictors of MTX intolerance or insufficient efficacy. This study aimed to investigate genetic variants associated with MTX intolerance or insufficient efficacy leading to therapy switch. **Methods:** A total of 80 patients with moderate-to-severe psoriasis were included: 43 who required switching from MTX to biologics and 37 who continued MTX therapy. Twelve polymorphisms in transporter and metabolism-related genes (*ABCB1* (rs1045642), *MTHFR* (rs1801133), *ABCB1* (rs1128503), *ABCC2* (rs3740066), *ABCC2* (rs717620), *ABCG2* (rs2231137), *GSTP1* (rs1695), *SLC19A1* (rs1051266), *COL18A1* (rs9977268), *SLCO1B1* (rs2306283), *SLCO1B1* (rs4149056), and *ABCB1* (rs2229109)) were analyzed using next-generation sequencing. **Results:** Significant differences in genotype frequencies were observed for *SLC19A1* rs1051266 (*p* = 0.03) and *COL18A1* rs9977268 (*p* = 0.02). Carriers of the T allele in both genes were more frequent among patients requiring biologic therapy, suggesting a possible association with MTX intolerance or reduced efficacy. **Conclusions:** The study revealed an association between polymorphisms in the *SLC19A1* rs1051266 and *COL18A1* rs9977268 genes and the need to switch from MTX to biologic therapy in patients with moderate-to-severe psoriasis. These findings suggest that carriers of the C allele in these genes may have an increased risk of methotrexate intolerance.

## 1. Introduction

Psoriasis is a chronic multifactorial disease characterized by accelerated keratinocyte proliferation and impaired differentiation, as well as an imbalance between pro- and anti-inflammatory cytokines, often accompanied by pathological changes in the musculoskeletal system. According to statistical data, the prevalence of psoriasis in the Russian Federation in 2024 was 262.8 cases per 100,000 population, with an incidence of 59.3 per 100,000 population [1]. The therapeutic strategy for psoriasis is determined by patient characteristics (age, sex, comorbidities) and disease features (type, localization, severity). Recent studies have highlighted the heterogeneity of disease mechanisms and emphasized the need for individualized treatment strategies, especially in patients with moderate-to-severe psoriasis requiring systemic therapy, particularly with methotrexate (MTX) [2].

The pharmacological effect of MTX is primarily due to its ability to inhibit multiple enzymes involved in de novo nucleotide biosynthesis, such as dihydrofolate reductase, thymidylate synthase, aminoimidazole carboxamide ribonucleotide transformylase, and amidophosphoribosyltransferase [3]. MTX can also be considered a prodrug, since within 24 h of administration, 95% of the dose undergoes polyglutamination, and its polyglutamates can be detected in erythrocytes long after the drug has been eliminated from plasma. The glutamate conversion is catalyzed by the enzyme folylpolyglutamate synthetase [4]. In addition, methotrexate polyglutamates act as inhibitors of 5-aminoimidazole-4-carboxamide ribonucleotide transformylase, leading to an intracellular increase in adenosine levels, a potent endogenous anti-inflammatory mediator [3,5].

Although MTX is a highly effective drug for the treatment of psoriasis, therapeutic outcomes are not always achieved and vary considerably among patients, which is apparently related to genetic predisposition to the response to this drug [6].

Currently, due to the absence of acitretin (Neotigason) in our country, MTX is used much more frequently for the treatment of severe psoriasis. In the Russian Federation, psoralen plus ultraviolet A (PUVA) therapy is still widely applied for severe psoriasis; however, it should be noted that long-term, repeated courses of phototherapy cause dose-dependent chronic photodamage to the skin. The most common manifestations include lentigo, diffuse hyperpigmentation, and actinic elastosis. Less frequently, reticular seborrheic keratosis, telangiectasias, and mottled skin pigmentation are observed. Since systemically administered psoralens can penetrate into the lens of the eye and, under UVA exposure, bind to lens proteins, PUVA therapy carries a potential risk of cataract development. Prolonged, repeated PUVA therapy also increases the risk of cutaneous squamous cell carcinoma [1]. As a result, the use of MTX for the treatment of severe psoriasis has increased significantly. However, physicians are often reluctant to prescribe MTX due to concerns about adverse effects.

The pharmacokinetics and pharmacodynamics of methotrexate are influenced by multiple factors, including drug transporters (e.g., SLC and ABC families), metabolic enzymes (e.g., MTHFR, GSTP1), and individual genetic variability among patients. The genes included in this study—*ABCB1*, *ABCC2*, *ABCG2*, *SLC19A1*, *SLCO1B1*, *MTHFR*, *GSTP1*, and *COL18A1*—encode proteins involved in methotrexate transport, metabolism may collectively affect drug efficacy and toxicity. Previous pharmacogenetic studies in psoriasis and rheumatoid arthritis have reported associations between MTX response and polymorphisms in SLC19A1, MTHFR, and ABC transporters, although the results have been inconsistent [7,8,9]. This variability underscores the importance of investigating these associations in ethnically distinct populations. Despite the availability of several studies addressing MTX pharmacogenetics in psoriasis, data remain limited and sometimes contradictory, particularly regarding the clinical need to switch from methotrexate to biologic therapy. Therefore, this study aimed to investigate pharmacogenetic predictors associated with this therapeutic transition in patients with moderate-to-severe psoriasis.

Currently, in cases where previous treatment approaches are ineffective—such as topical therapy (use of local corticosteroids, calcipotriol) or phototherapy (PUVA therapy), or in situations of intolerance or contraindications—selective immunosuppressants (phosphodiesterase-4 inhibitors, Janus kinase inhibitors) and biologic therapy (TNF-α inhibitors, IL-12/23 inhibitors, IL-17 inhibitors, IL-23 inhibitors) are employed [10,11]. Despite the availability of innovative treatment options with biologic agents, there is still a lack of studies assessing the relationship between gene polymorphisms and the need to switch from MTX to biologic therapy in patients with moderate-to-severe psoriasis.

Therefore, this study aimed to investigate pharmacogenetic predictors associated with this therapeutic transition in patients with moderate-to-severe psoriasis using targeted genotyping of key transporter- and metabolism-related genes. The analysis revealed significant associations for *SLC19A1* rs1051266 and *COL18A1* rs9977268 polymorphisms, which may contribute to interindividual variability in MTX efficacy and tolerance.

## 2. Materials and Methods

### 2.1. Ethical Approval

The study was approved by the Independent Ethics Committee of the Penza Institute for Advanced Medical Education—Branch of the Federal State Budgetary Educational Institution of Further Professional Education “Russian Medical Academy of Continuous Professional Education” of the Ministry of Healthcare of the Russian Federation (protocol number 25 dated 13 June 2024). The study was conducted in accordance with the legislation of the Russian Federation and international regulatory documents (the Declaration of Helsinki of the World Medical Association, 2013; National Standard of the Russian Federation GOST R 52379-2005).

All patients who participated in the study provided written informed consent. Before obtaining consent, the study protocol, potential risk factors, and other relevant aspects were explained in detail to each patient, who was also given the opportunity to ask questions.

### 2.2. Study Participants

The study included 80 individuals: 37 patients with moderate-to-severe psoriasis who had been receiving MTX for at least one year, and 43 patients with comparable disease severity who were switched from MTX to biologic therapy. The median ages were 56.0 [42.0; 67.0] and 51.0 [33.0; 62.0] years, respectively.

### 2.3. Clinical Assessment

Inclusion criteria were: a confirmed diagnosis of plaque psoriasis (L40.00) of moderate-to-severe severity with PASI > 10 and more than 20% of body surface area affected; and provision of written informed consent to participate in the study.

Exclusion criteria were: severe somatic pathology (liver, kidney, cardiovascular, respiratory, nervous system, or hematological diseases); psychotic state or a history of severe psychiatric disorders (schizophrenia, epilepsy, bipolar disorder, etc.).

All patients underwent standard physical and general clinical examinations, including collection of complaints and medical history, and evaluation of laboratory parameters: complete blood count and biochemical blood tests; urinalysis; pregnancy test (for women); and screening for viral infections (HIV, hepatitis B and C) and infectious diseases (syphilis). To assess psoriasis severity, the Psoriasis Area and Severity Index (PASI) and the Dermatology Life Quality Index (DLQI) were used. A PASI score of up to 10 indicates mild disease; 10–19, moderate; and 20–72, severe. The overall DLQI score was calculated as the sum of mean values from the individual questionnaire domains (scales). Clinical data were collected retrospectively from medical records. According to standard clinical practice, patients were routinely assessed at baseline (week 0), week 8, and week 24 to monitor treatment response and adverse events. These time points were used as reference visits for data extraction and analysis.

According to the British Association of Dermatologists’ Guidelines on the Safe Use of Methotrexate (2016), the criteria for discontinuing the drug include: total leukocyte count < 3 × 10^9^/L; neutrophil count < 1 × 10^9^/L; platelet count < 100 × 10^9^/L; MCV > 105 μL; elevation of AST or ALT to more than 2–3 times the reference values; onset or worsening of dyspnea or dry cough; severe sore throat; and unexplained bruising.

According to the Russian clinical guidelines, methotrexate therapy should be discontinued if the leukocyte count falls below 1.5 × 10^9^/L, the neutrophil count falls below 0.2 × 10^9^/L, or the platelet count falls below 75 × 10^9^/L, as well as in cases of increased serum creatinine or bilirubin levels. Discontinuation is also recommended in the event of diarrhea, ulcerative stomatitis, signs of pulmonary toxicity (particularly dry nonproductive cough), or signs of bone marrow suppression. Unusual bleeding or bruising, melena, hematuria, or petechiae require immediate medical consultation.

Both groups included patients who had been receiving systemic therapy for at least 12 months, ensuring comparable treatment duration for MTX and gene-engineered biologic therapy (GEBT). Differences in PASI scores between groups (median 22 [14; 30] for the MTX group vs. 30 [22; 38] for the GEBT group) were statistically significant and clinically meaningful, reflecting greater baseline disease severity among patients who later required biologic therapy.

The primary endpoint was defined as a categorical transition from MTX to GEBT (‘ever-switch’) during the clinical observation period. The primary reason for switching was categorized as either ‘lack of efficacy’ or ‘intolerance’ based on the clinical assessment documented at the time of the treatment change. The reasons for switching patients from MTX to biologic therapy were as follows: thrombocytopenia in 16.3% of cases, nausea in 11.6%, vomiting in 4.6%, leukopenia in 11.6%, and lack of efficacy in 74.4% of patients.

The clinical and laboratory characteristics of the examined patients are presented in Table 1.

To identify pharmacogenetic markers of methotrexate intolerance in patients with moderate-to-severe psoriasis, and based on the specialized PharmGKB database, the following gene polymorphisms were selected: *ABCB1* rs1045642, *MTHFR* rs1801133, *ABCB1* rs1128503, *ABCC2* rs3740066, *ABCC2* rs717620, *ABCG2* rs2231137, *GSTP1* rs1695, *SLC19A1* rs1051266, *COL18A1* rs9977268, *SLCO1B1* rs2306283, *SLCO1B1* rs4149056, and *ABCB1* rs2229109. The study was designed as an exploratory candidate-gene analysis; therefore, results were interpreted as hypothesis-generating.

ABC transporters (ATP-binding cassette, ABC) play a key role in many cellular and metabolic processes, as they transport a wide range of divergent endogenous substrates across plasma and intracellular membranes and contribute to protection against xenobiotic penetration [12,13].

Polymorphisms of the SLCO1B1 gene, which encodes the OATP1B1 (organic anion transporting polypeptide 1B1) protein, can significantly influence drug pharmacokinetics and determine individual variability in pharmacological response. According to the literature, SLCO1B1 polymorphisms may serve as predictors of hepatotoxicity [8,13].

Methylenetetrahydrofolate reductase (MTHFR) is a key enzyme in homocysteine/methionine metabolism that catalyzes the formation of 5-methyltetrahydrofolate, a donor of methyl groups for the synthesis of methionine from homocysteine. The 677 C>T mutation (rs1801133) in the *MTHFR* gene impairs its thermostability, leading to reduced enzymatic activity and elevated homocysteine levels. Literature data indicate that methotrexate treatment is associated with increased serum homocysteine concentrations depending on genotype [14].

The *COL18A1* gene encodes a heparan-sulfate-containing proteoglycan, collagen XVIII, from the C-terminal of which endostatin is released—a potent endogenous inhibitor of angiogenesis [15] that influences the course of psoriasis [16] and also acts as an initiator of redox signaling cascades [17].

GSTP1 is a member of the glutathione S-transferase (GST) superfamily, and its activity is largely determined by genotype. The *GSTP1* gene is located on chromosome 11q13 and is subject to polymorphism. A single nucleotide substitution A/G at rs1695 results in an isoleucine-to-valine change, which reduces substrate specificity, catalytic activity, and thermostability of the GSTP1 protein [18].

### 2.4. DNA Isolation and Genotyping

For molecular genetic analysis, blood samples were collected from the cubital vein into vacuum tubes with EDTA for subsequent DNA extraction and sequencing of coding regions within the target gene panel. Genomic DNA (gDNA) was isolated using the Blood DNA Mini Kit (Foregene, Chengdu, China). DNA concentrations were measured with the Qubit dsDNA HS Assay Kit on a Qubit 3.0 fluorometer (Thermo Fisher Scientific, Waltham, MA, USA). Library preparation for sequencing was performed using the KAPA HyperExome kit (Roche, Basel, Switzerland). Sequencing was carried out on an Illumina platform in 150 bp paired-end mode.

All exome samples were aligned to the reference genome assembly GRCh38.p13 using the Genome Analysis Toolkit (GATK) and the Burrows–Wheeler Aligner (BWA MEM, version 0.7.17; Wellcome Sanger Institute, Hinxton, UK). Variant calling was performed with GATK HaplotypeCaller (version 4.1.4; Broad Institute, Cambridge, MA, USA), followed by genotyping of the samples. Genetic variants were further filtered using GATK; genotypes with a total read depth of less than 10 were excluded. Filtered variants were annotated with Ensembl Variant Effect Predictor (VEP, version 103.1; European Bioinformatics Institute, Hinxton, UK), using data from the 1000 Genomes Project (phase 3), the Exome Aggregation Consortium, allele frequency data from Russian exomes, as well as NCBI ClinVar and dbNSFP v2.9 databases.

The following genes were included in the study: *ABCB1* (rs1045642), *MTHFR* (rs1801133), *ABCB1* (rs1128503), *ABCC2* (rs3740066), *ABCC2* (rs717620), *ABCG2* (rs2231137), *GSTP1* (rs1695), *SLC19A1* (rs1051266), *COL18A1* (rs9977268), *SLCO1B1* (rs2306283), *SLCO1B1* (rs4149056), and *ABCB1* (rs2229109).

### 2.5. Statistical Analysis

Statistical analysis was performed using STATISTICA 12.0 (Dell Statistica, Tulsa, OK, USA). For comparison of quantitative data, the nonparametric Mann–Whitney U test was applied. To assess differences between groups for qualitative variables, the χ^2^ test with Yates’ correction was used; in cases where test assumptions were not met, Fisher’s exact two-tailed test was applied. Statistical significance was set at *p* < 0.05. The strength of associations was estimated by odds ratios (OR) with 95% confidence intervals (CI).

All SNPs were analyzed under a prespecified dominant genetic model to minimize multiple testing. SNPs deviating from Hardy–Weinberg equilibrium were excluded from association analyses.

False discovery rate (FDR) correction was applied using the Benjamini–Hochberg procedure, and q-values are reported alongside nominal *p*-values.

Given the study’s retrospective design and the primary endpoint defined as a categorical group membership (MTX continuers vs. switchers), between-group comparisons for genetic associations were performed using univariate tests. We acknowledge that key clinical parameters (e.g., baseline PASI, disease duration) differed between groups. However, due to the limited sample size, which would lead to overfitting and unstable estimates, a formal multivariable regression model adjusting for these potential confounders was not performed. This analytical constraint is discussed as a limitation.

## 3. Results

The distribution of genotypes of the studied polymorphic gene variants was assessed for compliance with the Hardy–Weinberg equilibrium (HWE) (Table 2).

All investigated loci were tested for compliance with the HWE. Two *ABCB1* variants deviated from HWE (*p* < 0.05); such deviations may reflect technical artifacts or clinical selection effects. Therefore, both variants were excluded from subsequent association analysis.

The results of genotyping for the following allelic variants—*MTHFR* (rs1801133), *ABCB1* (rs1128503), *ABCC2* (rs3740066), *ABCC2* (rs717620), *ABCG2* (rs2231137), *GSTP1* (rs1695), *SLC19A1* (rs1051266), *COL18A1* (rs9977268), *SLCO1B1* (rs2306283), and *SLCO1B1* (rs4149056)—are presented in Table 3.

According to the results, statistically significant differences in genotype frequencies between the patient groups were observed for two SNPs: *SLC19A1* rs1051266 and *COL18A1* rs9977268. When assessing the distribution of genotypes for rs1051266 in the *SLC19A1* gene, statistically significant differences were observed between patients receiving MTX and those on biologic therapy (*p* = 0.03). Carriers of the T allele of *SLC19A1* rs1051266 were more frequently observed among patients who required switching to biologic therapy, suggesting that the T allele may be associated with reduced MTX efficacy. For rs9977268 in the *COL18A1* gene, CC genotype was more common among patients who continued MTX therapy, but carriage of the T allele was associated with a higher likelihood of switching to biologic therapy (OR = 3.21, *p* = 0.02), suggesting that this variant may contribute to methotrexate intolerance or reduced efficacy.

After Benjamini–Hochberg FDR correction, the associations for *SLC19A1* rs1051266 and *COL18A1* rs9977268 did not retain formal statistical significance (q = 0.15) and therefore should be interpreted as exploratory.

## 4. Discussion

In our study, the T allele of both *SLC19A1* rs1051266 and *COL18A1* rs9977268 was more frequently observed among patients who required switching to biologic therapy, suggesting that this variant may predispose to methotrexate intolerance or lower treatment efficacy. However, after FDR correction, the associations did not remain statistically significant; therefore, the results should be interpreted as exploratory.

The transmembrane protein SLC19A1 belongs to the solute carrier family and was previously known as the reduced folate carrier 1 (RFC1). It plays an important role in the intracellular transport of methotrexate [19]. To date, SNPs in the *SLC19A1* gene have been studied for their association with MTX-induced toxicity as well as treatment efficacy [20]. One of the most extensively investigated variants is rs1051266 [20]. This SNP is located in exon 2 of *SLC19A1* at position 80, where guanine can be replaced by adenine. As a result, histidine is substituted with arginine, leading to structural changes in the transporter protein and, consequently, altered functional activity [21]. Accordingly, *SLC19A1* modifies the affinity of the protein for methotrexate, which increases the amount of the drug remaining in the circulation [22].

*COL18A1* encodes the α1 chain of type XVIII collagen, with the gene located on chromosome 21q22.3. Proteolysis of this protein produces endostatin, a 20 kDa protein primarily found in hepatic sinusoids and basement membranes [23]. Studies have shown that endostatin can initiate the NADPH oxidase-dependent redox signaling cascade, leading to increased generation of reactive oxygen species and enhanced oxidative stress [17]. Elevated serum levels of endostatin have been observed in patients; however, the pathophysiological mechanisms underlying this phenomenon remain unclear [24]. Previously, rs9977268 was associated with a “poor” response to MTX [25].

The *SLC19A1* rs1051266 polymorphism (G>A) results in a histidine-to-arginine substitution that may reduce transporter affinity for MTX, potentially leading to decreased intracellular accumulation and diminished efficacy, as supported by previous pharmacogenetic investigations in rheumatoid arthritis and psoriasis [25,26]. For *COL18A1* rs9977268, emerging evidence suggests it may influence endostatin production or redox signaling, which could modulate inflammatory responses relevant to MTX tolerance, although direct functional data remain limited [7]. These mechanistic hypotheses warrant further investigation through targeted functional assays in future studies. Taken together, these data suggest that genetic variability in SLC19A1 may alter intracellular methotrexate availability, whereas changes in COL18A1 could influence tissue remodeling and inflammatory processes through altered endostatin-mediated signaling. Such combined pharmacokinetic and pharmacodynamic effects may partially explain the interindividual variability in treatment response and the need to switch to biologic therapy observed in our study.

In our study, the transition from methotrexate to biologic therapy occurred mainly due to insufficient therapeutic efficacy rather than toxicity. Specifically, 74.4% of patients in the GEBT group discontinued MTX because of a lack of response, whereas only a minority experienced adverse effects such as thrombocytopenia, leukopenia, or gastrointestinal intolerance. Therefore, the observed associations between genetic polymorphisms and the need to switch to biologics may reflect both reduced efficacy and potential intolerance to MTX. It is well known that pharmacological response to treatment is highly individualized and may be determined by alterations in DNA sequences (including single-nucleotide polymorphisms, deletions, insertions, etc.). Such changes in nucleotide sequences (polymorphisms, mutations) may impair or abolish the function of proteins that facilitate drug transport into cells, thereby reducing treatment efficacy and potentially triggering adverse drug reactions.

Our findings are in partial agreement with previous pharmacogenetic studies evaluating methotrexate response in psoriasis. Several reports have associated *SLC19A1* rs1051266 polymorphism with reduced MTX efficacy or increased toxicity, although results have varied across populations [8,25,26]. In contrast, the *COL18A1* rs9977268 variant has been less frequently studied; however, recent data suggest its possible involvement in oxidative stress-related pathways that may influence MTX response [7]. The discrepancies between studies likely reflect differences in sample size, ethnic background, clinical heterogeneity, and criteria for defining treatment response.

Several methodological aspects should be considered when interpreting these findings. First, the relatively small sample size (n = 80) limits the statistical power of the analysis and may affect the generalizability of the findings. In addition, certain baseline clinical characteristics differed between the two study cohorts. Patients who switched to biologic therapy (GEBT group) had a longer median disease duration (20.0 [14.0; 26.0] years) and higher baseline PASI scores compared with patients who continued methotrexate (MTX group; 14.5 [7.0; 20.0] years; *p* = 0.02). Due to the relatively small sample size, our analysis was unable to adjust for key clinical differences between the groups (such as higher baseline PASI and longer disease duration in the GEBT group) using multivariable models (e.g., logistic regression). These clinical factors are strong predictors of the need for advanced therapy and could confound the observed genetic associations. These differences likely reflect real-world clinical practice, in which patients with more severe and long-standing psoriasis tend to exhibit lower therapeutic response or intolerance to MTX, leading to the initiation of biologic therapy. The lack of adjustment means our reported associations (ORs) should be interpreted as unadjusted estimates. This clinical heterogeneity was taken into account during data interpretation and is acknowledged as a limitation of the study design. Second, the study population was recruited from a limited number of clinical centers in the Russian Federation, which may not fully reflect the genetic and clinical diversity of patients with psoriasis in other populations. Third, only a selected panel of polymorphisms in candidate genes was analyzed; therefore, potentially relevant genetic variants outside this panel may have been overlooked. Fourth, the cross-sectional design precludes assessment of causal relationships between genetic polymorphisms and the need to switch from methotrexate to biologic therapy. Fifth, more extensive genomic quality control and alternative genetic models were not explored due to the targeted sequencing design and limited sample size. Finally, environmental and clinical factors, such as adherence to treatment, concomitant medications, and comorbidities, were not comprehensively evaluated. Future studies with larger, multi-center cohorts, extended genomic analysis, and longitudinal follow-up are required to validate and expand upon our initial findings.

## 5. Conclusions

Thus, this study demonstrated an association between polymorphisms in the *SLC19A1* rs1051266 and *COL18A1* rs9977268 genes and the need to switch from methotrexate to biologic therapy in patients with moderate-to-severe psoriasis. These findings suggest that carriers of the T allele in these genes may have an increased likelihood of methotrexate intolerance or insufficient treatment response.

Exploring potential genetic predispositions associated with the need for earlier switching to biologic therapy in patients with moderate-to-severe psoriasis may help to develop a personalized treatment approach for this patient group, aiming to reduce the risk of disability and improve quality of life.

## Figures and Tables

**Table 1:** Clinical and laboratory parameters in patients with moderate-to-severe psoriasis receiving methotrexate (MTX) or Gene-engineered biologic therapy (GEBT).

Clinical and Laboratory Parameter	Group 1 (MTX), n = 37	Group 2 (GEBT), n = 43	*p*-Value
Frequency of exacerbations, per year	2.0 [2.0; 2.0]	3.0 [3.0; 3.0]	<0.0001 *
Duration of disease, years	14.5 [7.0; 20.0]	20.0 [14.0; 26.0]	0.02 *
Psoriasis severity at the time of inclusion in the study, PASI index	22.0 [19.0; 33.0]	30.0 [25.0; 41.0]	0.0002 *
Hemoglobin, g/L	141.5 [128.0; 153.0]	151.0 [140.0; 157.0]	0.005 *
Erythrocytes, 10^12^/L	4.66 [4.29; 4.95]	4.8 [4.45; 5.2]	0.132093
ESR, mm/h	17.0 [7.0; 25.0]	15.0 [10.0; 22.0]	0.328210
Platelets, 10^9^/L	248.0 [211.0; 276.0]	207.0 [163.0; 261.0]	0.021422 *
Leukocytes, 10^9^/L	7.36 [6.1; 8.61]	6.39 [5.52; 7.8]	0.100253
ALT, U/L	25.15 [17.8; 42.2]	25.3 [20.0; 33.0]	0.691398
AST, U/L	25.95 [20.0; 39.4]	26.7 [23.0; 32.0]	0.914343
Total bilirubin, μmol/L	9.2 [6.6; 13.2]	11.0 [8.2; 13.8]	0.156293
Creatinine, μmol/L	76.6 [67.9; 86.8]	76.9 [67.2; 89.4]	0.867440
Glucose, mmol/L	5.61 [5.13; 6.55]	5.69 [5.3; 6.3]	0.702378
Total protein, g/L	72.25 [68.7; 75.6]	71.6 [69.6; 75.1]	0.855780

Note: *—Statistically significant *p*-values were observed; ESR—erythrocyte sedimentation rate; ALT—Alanine aminotransferase; AST—Aspartate transaminase; GEBT—Gene-engineered biologic therapy; MTX—methotrexate.

**Table 2:** Frequency of occurrence of polymorphic gene variants with respect to Hardy–Weinberg equilibrium.

Variant	Genotype		GEBT (n = 43)				MTX (n = 37)		
		Distribution, Absolute (Relative %)	MAF	χ^2^	*p*-Value	Distribution, Absolute (Relative %)	MAF	χ^2^	*p*-Value
*ABCB1* rs1045642	AA/AG/GG	13 (30.23)/24 (55.8)/6 (13.95)	41.9	0.9250	0.3362	7 (18.92)/25 (67.57)/5 (13.51)	47.3	4.6711	0.0307 *
*MTHFR* rs1801133	GG/GA/AA	17 (39.53)/23 (53.49)/3 (6.98)	33.7	1.6622	0.1973	14 (37.84)/16 (43.24)/7 (18.92)	40.5	0.3928	0.5309
*ABCB1* rs1128503	AA/AG/GG	11 (25.58)/21 (48.84)/11 (25.58)	50.0	0.0233	0.8788	12 (32.43)/17 (45.95)/8 (21.62)	44.6	0.1824	0.6693
*ABCC2* rs3740066	CC/CT/TT	19 (44.19)/20 (46.51)/4 (9.3)	32.6	0.1503	0.6983	13 (35.14)/18 (48.65)/6 (16.22)	40.5	0.0031	0.9559
*ABCC2* rs717620	CC/CT/TT	27 (62.79)/15 (34.88)/1 (2.33)	19.8	0.4278	0.5131	24 (64.86)/12 (32.43)/1 (2.7)	18.9	0.1208	0.7282
*ABCG2* rs2231137	CC/CT/TT	40 (93.02)/3 (6.98)/0 (0)	3.5	0.0562	0.8126	30 (81.08)/7 (18.92)/0 (0)	9.5	0.4039	0.5251
*GSTP1* rs1695	AA/AG/GG	27 (62.79)/12 (27.91)/4 (9.3)	22.1	2.0469	0.1525	20 (54.05)/15 (40.54)/2 (5.41)	25.7	0.1432	0.7052
*SLC19A1* rs1051266	TT/TC/CC	8 (18.6)/25 (58.14)/10 (23.26)	52.3	1.1751	0.2784	15 (40.54)/13 (35.14)/9 (24.32)	41.9	2.8661	0.0905
*COL18A1* rs9977268	CC/CT/TT	34 (79.07)/7 (16.28)/2 (4.65)	12.8	3.1417	0.0763	20 (54.05)/16 (43.24)/1 (2.7)	24.3	1.128	0.2882
*SLCO1B1* rs2306283	AA/AG/GG	16 (37.21)/16 (37.21)/11 (25.58)	44.2	2.594	0.1073	18 (48.65)/13 (35.14)/6 (16.22)	33.8	1.7055	0.1916
*SLCO1B1* rs4149056	TT/TC/CC	31 (72.09)/11 (25.58)/1 (2.33)	15.1	0.0004	0.9835	31 (83.78)/5 (13.51)/1 (2.7)	9.5	1.6486	0.1991
*ABCB1* rs2229109	CC/CT/TT	42 (97.67)/0 (0)/1 (2.33)	3.4	43.00	5.4740 × 10^−11^ *	35 (94.59)/0 (0)/2 (5.41)	5.4	37.00	1.1813 × 10^−9^ *

Note: *—Statistically significant *p*-values were observed; GEBT—Gene-engineered biologic therapy; MTX—methotrexate; MAF—Minor Allele Frequencies.

**Table 3:** Frequency of polymorphic gene genotypes in patients with moderate-to-severe psoriasis according to therapy.

Variant	Genotype	Number of Patients				χ^2^	*p*-Value	FDR q-Value	OR	95% CI
		Absolute		In Fractions						
		MTX	GEBT	MTX	GEBT					
*MTHFR* rs1801133	GG	14	17	0.378	0.395	0.02	0.88	0.39	0.93	0.38–2.30
	GA+AA	23	26	0.622	0.605				1.07	0.44–2.65
*ABCB1* rs1128503	AA	12	11	0.324	0.256	0.46	0.50	0.37	1.40	0.53–3.69
	AG+GG	25	32	0.676	0.744				0.72	0.27–1.89
*ABCC2* rs3740066	CC	13	19	0.351	0.442	0.68	0.41	0.46	0.68	0.28–1.69
	CT+TT	24	24	0.649	0.558				1.46	0.59–3.61
*ABCC2* rs717620	CC	24	27	0.649	0.628	0.04	0.85	0.85	1.09	0.44–2.73
	CT+TT	13	16	0.351	0.372				0.91	0.37–2.28
*ABCG2* rs2231137	CC	30	40	0.811	0.930	2.59	0.11	0.37	0.32	0.08–1.35
	CT+TT	7	3	0.189	0.070				3.11	0.74–13.04
*GSTP1* rs1695	AA	20	27	0.541	0.628	0.63	0.43	0.43	0.70	0.28–1.71
	AG+GG	17	16	0.459	0.372				1.43	0.59–3.51
*SLC19A1* rs1051266	TT	15	8	0.405	0.186	4.67	0.03 *	0.15	2.98	1.09–8.19
	TC+CC	22	35	0.595	0.814				0.34	0.12–0.92
*COL18A1* rs9977268	CC	20	34	0.541	0.791	5.67	0.02 *	0.15	0.31	0.12–0.83
	CT+TT	17	9	0.459	0.209				3.21	1.21–8.54
*SLCO1B1* rs2306283	AA	18	16	0.486	0.372	1.06	0.30	0.37	1.60	0.65–3.91
	AG+GG	19	27	0.514	0.628				0.63	0.26–1.53
*SLCO1B1* rs4149056	TT	31	31	0.838	0.721	1.56	0.21	0.37	2.00	0.67–6.00
	TC+CC	6	12	0.162	0.279				0.50	0.17–1.50

Note: *—Statistically significant *p*-values were observed; GEBT—Gene-engineered biologic therapy; MTX—methotrexate; q-values calculated by the Benjamini–Hochberg false discovery rate (FDR); OR—odds ratio; CI—confidence interval.

## Data Availability

The datasets generated and analyzed during this study are not publicly available due to ethical restrictions and patient confidentiality protections under Russian Federation laws on personal data protection (Federal Law No. 152-FZ). However, anonymized data supporting the findings may be made available upon reasonable request from qualified researchers, subject to approval by the Independent Ethics Committee of the Penza Institute for Advanced Medical Education—Branch of the Federal State Budgetary Educational Institution of Further Professional Education “Russian Medical Academy of Continuous Professional Education” of the Ministry of Healthcare of the Russian Federation (protocol number 25 dated 13 June 2024) (contact: otd_nauka@piuv.ru). Requests should include a detailed research proposal and data protection plan.

## Abbreviations

The following abbreviations are used in this manuscript:

MTX	Methotrexate
PUVA	Plus ultraviolet A therapy
GEBT	Gene-engineered biologic therapy
MTHFR	Methylenetetrahydrofolate reductase
